# Fourier-Transform Infrared Imaging Spectroscopy and Laser Ablation -ICPMS New Vistas for Biochemical Analyses of Ischemic Stroke in Rat Brain

**DOI:** 10.3389/fnins.2018.00647

**Published:** 2018-09-19

**Authors:** Mohamed H. M. Ali, Fazle Rakib, Essam M. Abdelalim, Andreas Limbeck, Raghvendra Mall, Ehsan Ullah, Nasrin Mesaeli, Donald McNaughton, Tariq Ahmed, Khalid Al-Saad

**Affiliations:** ^1^Neurological Disorders Research Center, Qatar Biomedical Research Institute, Hamad Bin Khalifa University, Qatar Foundation, Doha, Qatar; ^2^Department of Chemistry and Earth Sciences, Qatar University, Doha, Qatar; ^3^Diabetes Research Center, Qatar Biomedical Research Institute, Hamad Bin Khalifa University, Qatar Foundation, Doha, Qatar; ^4^Department of Cytology and Histology, Faculty of Veterinary Medicine, Suez Canal University, Ismailia, Egypt; ^5^Institute of Chemical Technologies and Analytics, Vienna University of Technology, Vienna, Austria; ^6^Qatar Computing Research Institute, Hamad Bin Khalifa University, Qatar Foundation, Doha, Qatar; ^7^Weill Cornell Medicine-Qatar, Qatar Foundation, Doha, Qatar; ^8^Centre for Biospectroscopy, School of Chemistry, Monash University, Clayton, VIC, Australia

**Keywords:** photothrombotic, ischemic, brain, stroke model, FTIR imaging spectroscopy, LA-ICPMS, lipid peroxidation, neurodegeneration

## Abstract

**Objective:** Stroke is the main cause of adult disability in the world, leaving more than half of the patients dependent on daily assistance. Understanding the post-stroke biochemical and molecular changes are critical for patient survival and stroke management. The aim of this work was to investigate the photo-thrombotic ischemic stroke in male rats with particular focus on biochemical and elemental changes in the primary stroke lesion in the somatosensory cortex and surrounding areas, including the corpus callosum.

**Materials and Methods:** FT-IR imaging spectroscopy and LA-ICPMS techniques examined stroke brain samples, which were compared with standard immunohistochemistry studies.

**Results:** The FTIR results revealed that in the lesioned gray matter the relative distribution of lipid, lipid acyl and protein contents decreased significantly. Also at this locus, there was a significant increase in aggregated protein as detected by high-levels Aβ_1-42_. Areas close to the stroke focus experienced decrease in the lipid and lipid acyl contents associated with an increase in lipid ester, olefin, and methyl bio-contents with a novel finding of Aβ_1-42_ in the PL-GM and L-WM. Elemental analyses realized major changes in the different brain structures that may underscore functionality.

**Conclusion:** In conclusion, FTIR bio-spectroscopy is a non-destructive, rapid, and a refined technique to characterize oxidative stress markers associated with lipid degradation and protein denaturation not characterized by routine approaches. This technique may expedite research into stroke and offer new approaches for neurodegenerative disorders. The results suggest that a good therapeutic strategy should include a mechanism that provides protective effect from brain swelling (edema) and neurotoxicity by scavenging the lipid peroxidation end products.

## Introduction

Stroke is the leading cause of adult disability in the world, leaving more than half of those affected dependent on daily assistance. Patients are often hospitalized and, or subjected to intensive rehabilitation programs for long periods, with their quality of life severely affected, socially and economically. Hence, a more thorough understanding of the molecular and cellular changes are required for the design of better therapies to treat this debilitating illness (Ramasubbu et al., 1998; Sarti et al., 2000; Snögren and Sunnerhagen, 2009; Lekander et al., 2017).

Ischemic stroke is caused by a reduction in blood circulation in the brain vasculature, resulting in decreased supply of oxygen and nutrients followed by an ischemic cascade, and if not reversed results in neurodegeneration and ultimately cell death (Deb et al., 2010; Xing et al., 2012; Hu et al., 2017). Acute ischemic stroke causes unregulated cation influx, mainly Na^+^, which initiates the cytotoxic edema (Liang et al., 2007). Also, accumulation of Ca^2+^ inside forebrain neurons initiates glutamate mediated excitotoxicity (Lai et al., 2014; Prentice et al., 2015). This enhanced intra-cellular calcium-levels activates numerous pathways including proteases which can degrade proteins and membrane lipids and promote the generation of free radicals and reactive oxygen species (ROS) which can lead to neuronal damage, particularly of the cell membrane (Lee et al., 2000; Bretón and Rodríguez, 2012). Furthermore, mitochondria dysfunction can also result in apoptosis (Broughton et al., 2009; Xing et al., 2012). Stroke insult, also weakens and disturbs the blood–brain barrier (BBB) which can result in the development of vasogenic edema (Dostovic et al., 2016). Vasogenic edema causes disturbances in brain function, mass-effect (displacing surrounding brain tissues) with distortion, tissue shift and increased intracranial pressure and damage (Simard et al., 2007; Michinaga and Koyama, 2015).

Tissue in adjacent regions also undergo morphological, cellular and sub-cellular changes (Dirnagl et al., 1999; Lo et al., 2003; Shichita et al., 2014; Hu et al., 2017), such as gliosis (Garcia, 1984; Li et al., 2005; Huang et al., 2014), lipid peroxidation (Yamamoto et al., 1983; Yildirim et al., 2007; Zeiger et al., 2009) and protein malformation (Brouns et al., 2010; Zhao and Bateman, 2015). Perilesional changes are not necessarily detrimental, as neuronal plasticity and tissue reorganization in GM and WM may compensate and contribute to recovery post ischemic stroke (Carmichael, 2003; Cheatwood et al., 2008; Murphy and Corbett, 2009). Delay and ongoing tissue damage post ischemic stroke results in impeding brain tissue repair and recovery and contributes to brain dysfunctions. A model to study ischemic stroke is the photo-thrombotic model of focal ischemia, where photosensitive dye is excited by fluorescence causing a blood clot (Pevsner et al., 2001). Conventional techniques, such as magnetic resonance imaging (MRI) (Van Bruggen et al., 1992; Pevsner et al., 2001), auto-radiography (Dietrich et al., 1986), routine histology (Van Bruggen et al., 1992; Pevsner et al., 2001; Kuroiwa et al., 2009), and IHC (Van Bruggen et al., 1992) have been applied to characterize the biochemical and anatomical alterations after stroke in many studies (Dietrich et al., 1986; Pevsner et al., 2001; Kuroiwa et al., 2009). These techniques provide important data, but for example, MRI is not sufficient to study bio-chemical and bio-molecular changes at cellular and sub-cellular levels. Whereas, histology and IHC give high spatial resolution but again, only limited to certain biochemical markers as well as disrupting the tissue morphology.

Spectroscopic techniques such as FT-IR imaging spectroscopy and laser ablation inductively coupled plasma mass spectrometry (LA-ICP-MS) are potentially attractive bio-imaging platforms that can provide molecular (Kazarian and Chan, 2006; Petibois and Desbat, 2010) and elemental images (Becker et al., 2014), respectively. Both techniques offer high resolution (in the range of 5–10 μm), yielding detailed information about bio-chemical and chemical features at the cellular and/or sub-cellular level (Lasch et al., 2002; Kumar et al., 2014; Harrison and Berry, 2017) related to pathology. In contrast to standard histological staining methods, FTIR imaging has the ability to detect, simultaneously, discrete changes in molecular structure and composition of tissues. Direct biochemical analyses of all macromolecular components within tissue samples can be obtained from a single data acquisition, without the addition of chemical stains or reagents and without disrupting the tissue morphology (Petibois et al., 2009; Miller et al., 2013). Infrared (IR) spectra are dominated by macromolecular building blocks, such as proteins, lipids, cholesterols, phospholipids, carbohydrates, and nucleic acids (Carter et al., 2010; Srinivasan, 2010; Ami et al., 2013). The IR spectral absorptions provide readouts about these bio-molecules and produce a specific biochemical fingerprint of the sample (Ami et al., 2013; Baker et al., 2014; Kohler et al., 2015). FT-IR based techniques have been used to image molecular alterations associated with neuro-degeneration in animal models of Alzheimer’s disease (AD) (Miller et al., 2006; Leskovjan et al., 2010; Liao et al., 2013; Benseny-Cases et al., 2014), Parkinson’s disease (PD) (Szczerbowska-Boruchowska et al., 2007), amyotrophic lateral sclerosis (Kastyak et al., 2010), multiple sclerosis (Heraud et al., 2010), cerebral malaria (Hackett et al., 2015b), epilepsy (Turker, 2012; Turker et al., 2014) and hemorrhagic stroke (Ali et al., 2016; Caine et al., 2016; Balbekova et al., 2017). These studies have shown the potential of FTIR to assess molecular and neurochemical changes associated with brain pathology.

Laser ablation inductively coupled plasma mass spectrometry (LA-ICP-MS) has been developed and established as a technique in the generation of quantitative images of metal distributions in thin brain tissue sections with applications in research related to neurological disorders. This technique detects trace elements that are essential for brain function, such as zinc (Zn), copper (Cu), and iron (Fe) (Becker et al., 2014). Simultaneous analysis of these trace elements in brain can yield important information on the processes of neuronal (dys) function for example after PD (Hare et al., 2009; Hare et al., 2010; Matusch et al., 2010, 2012), AD (Matusch and Becker, 2012), stroke (Becker et al., 2011) and brain tumor (Becker et al., 2011). LA-ICP-MS is suitable for the imaging of metals in thin cross sections of soft and hard biological tissues with very low concentrations such as sub μg/g level (Hare et al., 2009; Becker et al., 2011; Matusch and Becker, 2012; Becker et al., 2014). The advantages of LA-ICP-MS is that it is highly sensitivity, accurate, precise as well as being a unique technique to scan the elemental distribution in the whole brain section.

In the present study, we combined FTIR and LA-ICP-MS with routine histological/IHC to characterize the bio-chemical and elemental changes in PS-GM, PL-GM, L-WM, CL-WM, CL-GM, and CC following 1-week photothrombotic stroke in rat brain sections. Our goal was to use FTIR and LA-ICPMS imaging techniques with conventional histological studies to identify alterations of specific bio-molecules and elements associated with ischemic stroke. In addition, this study will advance stroke bio-diagnostics and treatment and provide links between tissue injury, plasticity and repair, as well as identifying novel therapeutic targets and interventions.

## Materials and Methods

### Animal Model

All experimental protocols and animal handling procedures were in accordance with the National Institutes of Health (NIH) recommendations and with guidelines of the European Communities Council Directive. The experimental animals protocol was approved by the by the University of Utrecht Animal Research Ethics Board (DEC 2013.I.08.063), and adhered to the Netherlands Council on Animal Care guidelines for humane animal use (Ali et al., 2016; Balbekova et al., 2017). A total of 12 male (11 week-old, Sprague-Dawley rats; Charles River Laboratories International, Wilmington, MA, United States), from which six animals were used in the Photothrombotic stroke, which was induced in the right sensorimotor cortex (Ali et al., 2016) and remainder six rats served as age-matched controls. Data were analyzed by comparing the lesioned generated values with the unlesioned (contralateral) hemisphere.

### Block and Tissue Preparation

One week after stroke induction, the animals were sacrificed with an overdose of isoflurane followed by transcardial perfusion. Brains were extracted, fixed in 4% paraformaldehyde (PFA) for 24 h and embedded in paraffin (Balbekova et al., 2017). Paraffin embedded tissue blocks were serially cut into 5 μm slices using a microtome (Leica RM 2155 semi-automated rotary microtome, Germany). Thin coronal sections of brain were taken from central location of the lesion (Bregma 1.20 mm). Serial and consecutive sections were obtained for FTIR measurements, LA-ICPMS, and hematoxylin and eosin (H&E) staining and IHC studies. The brain sections for FTIR imaging were mounted on a CaF_2_ window and a transflective slide. The brain sections used for the histology and IHC were mounted on Flex IHC microscopic slides (Dako, Fisher Scientific, United States). Routine histology was performed on control healthy rats to confirm normal tissue morphology (Ali et al., 2016).

### Immunohistochemistry

Slides of brain sections 5 μm sections were washed with phosphate buffered saline (PBS) and maintained in 0.1 M PBS with 0.2% Triton X-100, pH 7.4 (PBST) for 2 days at 4°C. For antigen retrieval, the slides were incubated in 10 mM sodium citrate containing 0.05% Tween 20, pH 6.0 for 1 h at room temperature. To block non-specific protein binding, sections were incubated in 6% bovine serum albumin (BSA) in PBST for 2 h at room temperature, then incubated for 3 days at 4°C with the primary antibodies. The primary antibodies (all Abcam, Ltd., Cambridge, United Kingdom, unless stated) were rabbit polyclonal anti-amyloid precursor protein (APP) (1:1000 dilution; ab2072), rabbit polyclonal anti-Tau (1:1000 dilution, ab47579), rabbit anti-GFAP (1:2000 dilution; ab7260), rabbit anti-MAP2 (1:2000 dilution; ab5622), or mouse anti-myelin basic protein (MBP) (1:2000 dilution; ab62631), or chicken anti-beta amyloid (1:2000 dilution; ab134022). The primary antibodies were diluted in 2% BSA in PBST. After the sections had been rinsed three times with PBST (10 min each), sections were incubated for 2 h at room temperature with Alexa Fluor 488-conjugated donkey anti-chicken IgG, Alexa Fluor 647-conjugated donkey anti-rabbit IgG, or Alexa Fluor 647-conjugated donkey anti-mouse IgG (1:500; Thermo Fisher Scientific, United States). Negative controls were subjected to the same protocol without primary antibodies. After three rinses with PBST, the mounting media containing DAPI (nuclear stain) were added to the slides and then overlayed with cover slips. The slides were kept away from light and examined using a fluorescence microscope.

### Fourier Transform Infrared Spectroscopy (FTIR)

Fourier transform infrared spectroscopy (FTIR) spectra were recorded using an Agilent FTIR Cary 620 micro-spectrometer in reflection mode within the range of 4000–700 cm^-1^ with 64 scans per spectrum, 4 cm^-1^ spectral resolution and spatial resolution 80 μm. Background single beam spectra were measured on a substrate without biological tissue by co-adding 64 scans. FTIR chemical images were recorded using a 64 × 64 Mercury Cadmium Telluride (MCT) Focal Plane Array (FPA) liquid nitrogen cooled detector in mosaic mode. FTIR imaging was performed with a 15× objective (numerical aperture = 0.62), yielding a pixel size of 5.5 μm × 5.5 μm. The average size of the brain is 1 cm × 1.2 cm. Three sections of the same anatomy per animal were examined. From each region of interest an area of 200 μm × 200 μm were analyzed. To ensure consistency in the data and negate technical errors, exemplar samples were mounted on CaF_2_ slides and measured in transmission mode as well as in the *trans*-reflective mode using transflective slides. In both modes, we obtained very similar results. To circumvent the electric field standing wave effect (EFSW) reported for variations in section thickness, we repeated exemplar sections at 8 and 10 μm thickness, and here again, we obtained very similar spectral profiles as the measured samples. **Supplementary Table S1** (Stable 1) shows the FTIR assignments used in the medical and biomedical fields (Goormaghtigh, 2016).

### Data Pre-processing and Analysis

All data processing and image generation were using either Resolution Pro. Software, (version 5.0), or Cytospec, (version 2.00.03) and Origin software (version 8). The representative FTIR spectrum acquired from brain WM region of a control rat brain in the spectral range of 4000–800 cm^-1^ is shown in **Supplementary Figure S1** and the detailed spectral band assignments are given in **Supplementary Table S1** in which different contribution to each band were presented (Kneipp et al., 2000; Cakmak et al., 2012). Baseline correction was performed on the full range of wavelengths for the spectra. FTIR images of the functional groups were calculated using the baseline areas under the FTIR absorption bands based on the assignments in (**Supplementary Table S1**) (Kneipp et al., 2000; Cakmak et al., 2012). In this study, the area under the curve from specific bands were calculated in order to measure different bio-chemical contents after photothrombotic ischemic stroke induction and values were normalized to the contralateral (unlesioned) hemisphere values. For example the total lipid was represented by (C–H stretching region) in the spectral range of 3000–2800 cm^-1^; lipid acyl ν_s_(CH_2_) groups at 2860–2840 cm^-1^; lipid ester ν(C = O) at 1755–1715 cm^-1^; olefinic = CH band at 3027–3000 cm^-1^; CH_3_ asymmetric stretching ν_as_(CH_3_) (methyl concentration) at 2960–2950 cm^-1^; amide I band (total protein) at 1700–1600 cm^-1^; β-sheet aggregate at 1630 cm^-1^ and α-helix structures at 1655 cm^-1^ as detailed in **Table 1** and **Supplementary Table S1** (Kneipp et al., 2000; Cakmak et al., 2012).

**Table 1:** The spectral regions used for analytical purposes.

Infrared band	Integrated spectral range (cm^-1^)
**Lipid components**	
CH_2_ symmetric stretching	2852–2800
CH_2_ asymmetric stretching	2915–2930
CH_3_ asymmetric stretching	2950–2960
^∗^C-H stretching	2994–2800
Olefin = CH	3000–3027
Carbonyl ester (C = O) stretching	1745–1731
**Protein components**	
Amide I	1700–1600
Amide II	1555–1535
β-sheet aggregate	1630
α-helix structures	1655

*^∗^Total lipid regions*.

The C-H spectral region is dominated by lipid contribution and a small contribution from proteins, carbohydrates and nucleic acids (Kneipp et al., 2000). Based on this understanding, the following CH_2_ asymmetric, CH_3_ asymmetric; olefinic = CH and lipid ester (C = O) lipid assignment were all good approximate values. Lipid molecular and structural alterations were investigated by evaluating the integrated area under specific lipid spectral bands as follows: CH_2_ asymmetric (chain lipid length); CH_3_ asymmetric (methyl concentration); olefinic = CH (unsaturated lipid) and lipid ester (C = O) (oxidative stress by-products) as shown in **Table 1**. Raw spectra were vector normalized and second derivative was computed by calculating forward difference twice and smoothed using spline fitting. The spline fitting used cubic spline interpolation of the second derivative to smoothen spectra. Resonance Mie scattering can affect the spectra from the border between a brain tissue and a substrate which is commonly existing in biological samples and spectra acquired from a substrate apart from brain tissue area were eliminated from analysis (Bassan et al., 2010). The contribution of different types of proteins was calculated by fitting a linear mixed model of Gaussian bands centered at the wavenumbers of the protein types. Relative distribution of β-sheet aggregate protein were determined at 1625 cm^-1^ from the second-derivative intensity spectrum (Pribic, 1994; Hackett et al., 2015b; Caine et al., 2016). The relative amount of the protein aggregation content was quantified from the curve fitting of original absorbance spectra (Hackett et al., 2015b). Curve fitting was performed with MATLAB (version 2014a, United States) over the spectral range 1700-1600 cm^-1^.

### Chemometric Data Pre-processing and Analysis

Chemometric analyses, incorporating principal component analysis (PCA) and hierarchical cluster analysis (HCA), were used to differentiate the spectral types with the FTIR spectra of the brain pre-processed prior to analysis using shape-preserving piece-wise cubic interpolation (Pribic, 1994). The infrared spectra were converted from Agilent system format to comma separated values (CSV) to create data sets that were suitable for PCA analysis using the “prcomp” function in the “stats” package in R (Becker et al., 1988; Sanchez, 2011). Three principal components (PCs) were used for analysis since they explained the highest percentage of variations. The 3D scores plot of the PCs that explain the majority of the variance in the dataset enabled spectra to be grouped according to the chemical information they contained (Pribic, 1994).

Hierarchical cluster analysis was applied using the A2R package in R to compare the three brain regions (PS-GM, PL-GM, and CL-GM) based on their PCA projections in the 3D score space (Becker et al., 1988; Pribic, 1994; Sanchez, 2011). HCA was used to group spectra that displayed the same degree of similarity by calculating the Euclidean distance between all the data sets using Ward’s algorithm. The result was visualized in a dendrogram and the grouping of the three brain regions were presented as images consisting of colors clusters according to the heterogeneity scale (Becker et al., 1988; Sanchez, 2011).

### Laser Ablation ICPMS

An ICPQMS (Agilent 7700) laser ablation system (CETAC Technologies, Omaha, NE, United States) with a Nd:YAG laser at wavelength 266 nm was used throughout this work. **Table 2** shows the laser ablation and ICP triggering operational conditions. The data were processed to generate elemental images using a macro, and image processing software ‘ImageJ.’ The scan speed of each line was 120 μms^-1^, and the ICP-MS was configured in data-only mode to collect 185 readings per line scan at a rate of 2 s^-1^ (Hare et al., 2010; Matusch and Becker, 2012).

**Table 2:** Parameters for measuring a brain samples by LA-ICPMS.

Parameters	Condition
Spot size	100 microns
Space between lines	4 micron
Laser energy	15%
Laser shot frequency	20 Hz
Shutter delay	10 s
Scan rate	160
Number of lines	120
Integration time	Varies from 0.02–0.06 s
Total integration time	0.614 s
Total acquisition time	67 s

### Statistical Analysis

A paired *t*-test was performed to assess significant differences between the molecular content of the PS-GM and PL-GM; PS-GM and CL-GM and PL-GM and the CL-GM tissue. For each group, a mean and standard deviation was generated for each animal. Then the individual animal values were averaged for each group. Mann–Whitney-*U* test was performed on all data to test for significant differences (*p* < 0.05) between control and treated groups. All *t*-tests were two tailed and the 95% confidence limit was used to test for significance.

## Results

### Histological and Immunohistochemical Changes After Photo-Thrombotic Stroke

**FIGURE 1:**
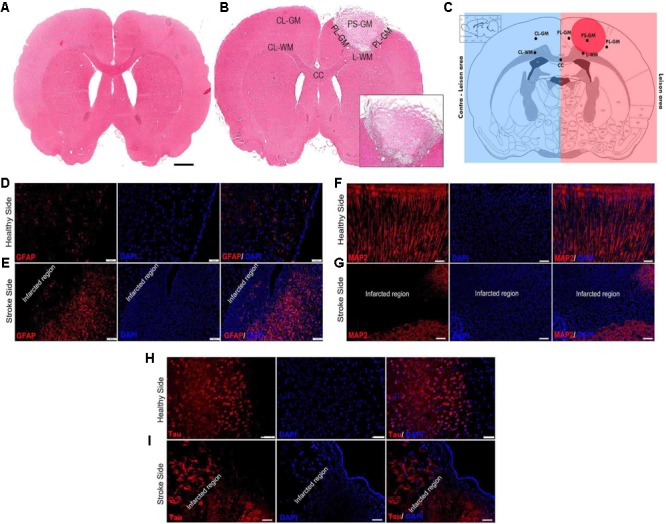
Photothrombotic lesion in rat somatosensory cortex results in cell death and astrigliosis. **(A)** Hematoxylin and Eosin (H&E) staining brain section of native control brain (magnification 5×). **(B)** H&E stained image 1-week post-stroke brain (magnification 5×). **(C)** Brain section scheme for the six regions of interest (ROI): primary stroke lesion gray matter (PS-GM), perilesional gray matter (PL-GM), lesioned white matter (L-WM) and contra-lesioned white matter (CL-WM), contra-lesioned gray matter (CL-GM), and corpus callosum white matter (CC). **(D,E)** Healthy control and lesioned brain sections labeled with GFAP and DAPI: primary lesion gray matter (PS), perilesional gray matter (PL) indicated activated astrocytes (GFAP, red stain) around the ischemic region (peri-infarct region) and degenerated neurons with shrunken nuclei (DAPI, blue stain). Infiltration of the astrocytes around the primary lesion PS-GM region with GFAP (red). The DAPI labels (blue) degenerated cells. **(F,G)** Healthy control and affected stroke rat brains sections were labeled with MAP2 and DAPI. **(H,I)** Healthy control and affected stroke rat brains sections labeled with Tau and DAPI to assist in comparisons. Scale bar = 100 μm.

One of the major aims of this study was to correlate molecular alterations after ischemic photo-thrombotic stroke, by using FTIR spectroscopy and elemental distribution changes (LA-ICPMS bio-imaging) and complement these results with immunostaining analyses of specific biomarkers. Hematoxylin and eosin (H&E) staining of the brain sections from unlesioned rats (native time-matched controls) is presented in **Figure 1A**. The locus of the photo-thrombotic ischemic stroke was confirmed using H&E staining (**Figure 1B**). The image clearly depicts loss of brain structure and integrity at the stroke focus and results in an absence of H&E staining within the ischemic infarct. The rim of the stroke region is the characteristic pale expression of the H&E staining due to brain edema (see inset **Figure 1B**). The contralateral hemisphere of the affected brain shows H&E strong staining expression. The six ROI for this study were: (a) the PS-GM; (b) PL-GM; (c) L-WM; (d) CL-WM; (e) CL-GM; and (f) CC (**Figure 1C**).

**FIGURE 2:**
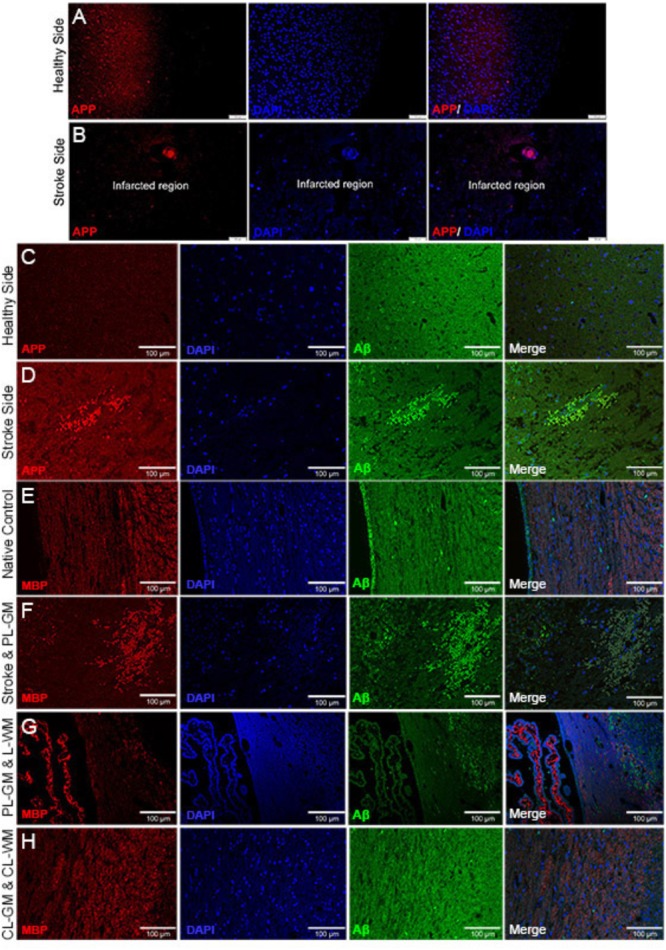
Immunohistochemistry staining identifying altered APP and Aβ_1-42_ in infarcted brain sections. **(A,B)** Contra-lesioned and lesioned hemispheres labeled with APP and DAPI: primary lesion gray matter (PS) shows amyloid precursor protein (APP) and contra-lesioned gray matter (CL-GM) with normal homogenous distribution of APP (red). **(C,D)** Contra-lesioned and affected stroke hemispheres were labeled with APP (red), DAPI (Blue) and Aβ_1-42_ (green). **(E–H)** Native control and 1-week affected stroke rat brains sections labeled with myelin basic protein (MBP, red), DAPI (blue), and Aβ_1-42_ (green). **(F,G)** Primary lesion gray matter (PS-GM) contains degenerated neurons (DAPI, blue) and disorganization of the myelin sheath (MBP, red) of the axonal neurons in the lesioned white matter (L-WM) scale bar = 100 μm for **(A–H)**.

To gain insights into the effects of stroke on nerve cells in the lesion as well as the healthy areas of the brain, IHC analyses using specific biomarkers was carried out. Immunohistochemical analysis of lesioned tissue sections (ipsilateral side) established neuronal degeneration in the infarct region with immunofluorescence analyses highlighting GFAP expression, the astrocyte marker, strongly localized within the PL-GM, indicating astrogliosis post stroke (**Figures 1D,E**). The infract region experienced neuronal loss, sections were tested for the principal neuronal marker proteins: MAP2 and Tau (**Figures 1F–I**). In both instances, we failed to detect these proteins within the infarct region- validating neuronal loss. Comparing the contralesion sections with the ischemic brain sections, we documented APP (**Figures 2A–D**) and aggregated Aβ_1-42_ (**Figures 2C–H**) in the GM and the PL-GM. Mis-processed APP, (Aβ_1-42_) its extracellular aggregation is one neuropathological marker used to classify Alzheimer’s disease (AD) (Paul Murphy and LeVine, 2010; Esparza et al., 2016; Wildburger et al., 2017). Surprisingly in the L-WM there was an increase in Aβ_1-42_ aggregates (**Figure 2G**). Examination of MBP showed loss in integrity and significant disorganization of the myelin sheaths in the WM, at the ipsilateral side, below the stroke point (**Figure 2G**). Interesting to note, that the contra-lesioned white matter (CL-WM, **Figure 2H**) shows uniform MBP staining throughout WM tracts as seen with the native control (**Figure 2E**).

### Molecular and Elemental Changes Due to Stroke

#### FTIR Spectra and Images

Fourier transform Infrared bands assignments in this study were based on the specific spectral bands as defined in the Section “Materials and Methods” (**Supplementary Figure S1** and **Supplementary Table S1**). As an example, the FTIR spectrum for the WM we observed that spectral bands corresponding to lipid, protein, ester, nucleic acids, and carbohydrates dominate the spectra (**Supplementary Figure S1**). All samples were prepared, treated and processed in the same manner, it should be noted that lipid, protein and other biochemical component values from the lesioned (ipsilateral side) were normalized to the contra-lateral side. Again, the contralateral hemisphere bio-chemical components values were used as a base line values indicating the stroke induced changes to the cortical region and the surrounding WM.

**FIGURE 3:**
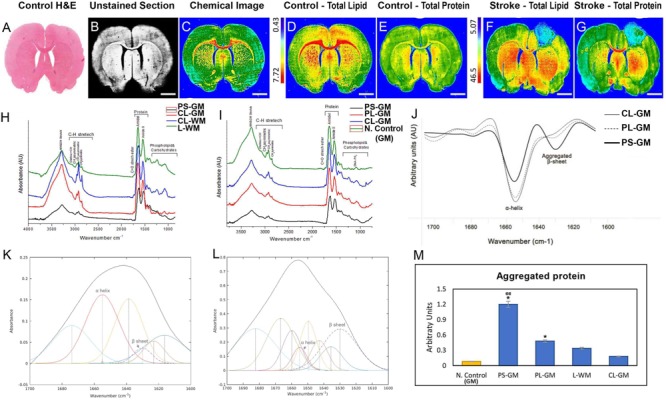
Whole brain section-FTIR imaging of biochemical changes within contralateral and ipsilateral hemispheres following photothrombotic focal ischemic insult to the somatosensory cortex. **(A)** H&E stained brain section captured by Digital Scanning Microscope Bright field (20×). **(B)**. Representative unstained FTIR light microscopic image of the healthy control rat brain (10×). **(C)**. Representative FTIR chemical image of the unstained brain section showing main biochemical components: lipid, phospholipid, protein, carbohydrates and nucleic acids. **(D,E)** FTIR image that represent the total lipid and total protein distribution in the healthy control rat brain. **(F,G)** FTIR image that represent the total lipid and total protein distribution in the stroke affected rat brain. Scale bars = 100 μm. **(H)** Representative FTIR averaged spectra in the range of 4000–400 cm^-1^ acquired from the cortical region of the PS-GM, L-WM, CL-GM, and CL-WM. **(I)** Representative FTIR averaged spectra in the range of 4000–400 cm^-1^ acquired from the cortical region of the PS-GM, PL-GM, CL-GM and native control GM. **(J)** Representative average second-derivative spectra of the amide I band in the spectral range of 1700–1600 cm^-1^ acquired from the primary lesion gray matter (PS-GM), perilesional gray matter (PL-GM), and contra-lesioned gray matter (CL-GM) regions post ischemic. The spectra show α- helical secondary protein structure at 1655 cm^-1^ and β-sheets protein conformation at 1630 cm^-1^. **(K,L)** Representative curve fitting of the amide I band in the spectral range of 1700-1600 cm^-1^ of the CL-GM and PS-GM, respectively, to quantify the aggregated protein relatively. **(M)** Histogram of the aggregated protein comparing different regions of interest: native control, PS-GM, PL-GM, L-WM with CL-GM. The FTIR images were colored-coded: red color corresponds to the highest content and blue color corresponds to the lowest content as shown on the color bars in the figures. Statistical significance was determined from six animals with a paired *t*-test and the 95% confidence interval. Bars represent mean ± SD. ^∗^*p* > 0.05, ^∗∗^*p* > 0.01 relative to CL-GM. ^σ^*p* > 0.05, ^σσ^*p* > 0.01 relative to PL-GM.

In Hematoxylin and Eosin section captured by digital scanning microscope, a parallel-unstained image of a control brain section, captured by FTIR microscope bright field is depicted together with the corresponding chemical FTIR spectroscopic image (**Figures 3A–C**). Lipid and protein distribution, as found in native healthy rat brain sections, was represented in **Figures 3D,E**. In contrast to the even distribution of protein and lipid in the control, the lesioned brain lipid and protein show distinct distribution patterns (**Figures 3F,G**). For example, in the PS-GM both lipid and protein levels were greatly reduced and associated with increasing aggregated protein. The PL-GM and L-WM regions experienced decrease in the lipid and protein components associated with protein aggregation (**Figures 3F,G** and see also **Figure 3M**). **Figures 3H,I** depict the average FTIR spectra for different tissue regions and the analysis of these spectra were performed on *n* = 6 animals. In the stroke PS-GM region, the FTIR spectra revealed that the lipid, protein, lipid acyl bands ν_s_(CH_2_), the lipid ester band ν(C = O) decrease compared with the PL-GM and CL-GM (**Figures 3H,I**). The stroke PS-GM FTIR spectrum had a significant reduction in the absorption band centered at 1227 cm^-1^ which arises from the P = O symmetric stretching vibrations of the phosphodiester bonds in DNA/RNA polysaccharide backbones (Carter et al., 2010; Srinivasan, 2010; Ami et al., 2013).

A more detailed representation of the average FTIR spectra of the PS-GM, CL-GM, CL-WM, and L-WM shown in **Figure 3H**. This highlights that lipid acyl bands were severely reduced in PS-GM and L-WM compared with CL-GM and CL-WM, respectively. The CH_3_ (methyl concentration), olefinic = CH (unsaturated lipid) and the lipid ester (CO) (oxidative stress) were significantly increased while that of the CH_2_ (chain lipid length) was decreased in the L-WM spectrum. The average FTIR spectra collected from the PS-GM, PL-GM, CL-GM and native control GM revealed marked reduction in lipid, lipid-acyl and protein in the ipsilateral compared with contralateral hemisphere; which were reduced when compared with the native control (**Figure 3I**).

**FIGURE 4:**
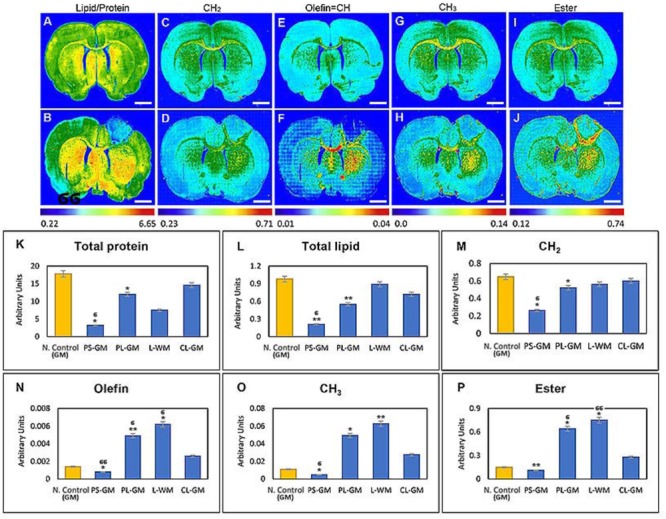
Functional group (macromolecular/sub-cellular/biochemical) images obtained of the native control ischemic stroke brain sections. **(A–I)**. Representative FTIR images of lipid/protein, lipid acyl group (CH_2_), olefin = CH, methyl (CH_3_) and lipid ester (C = O) distribution in the native healthy control brain tissue sections, respectively. **(B–J)** The same function groups (above) obtained from the ischemic stroke brain sections. **(K–P)** Histogram of specific bio-molecules content such as total protein, total lipid, lipid acyl group (CH_2_), olefin = CH, methyl (CH_3_), and lipid ester (C = O), respectively. The regions of interest were native control rat brain GM and ischemic brain PS-GM, PL-GM, L-WM, and CL-GM. The FTIR images were color coded, red color corresponds to the highest content and blue color corresponds to the lowest content as shown on the color bars in the figures. Statistical significance was determined from six animals. Statistical significance was determined from six animals with a paired *t*-test and the 95% confidence interval. Bars represent mean ± SD. ^∗^*p* > 0.05, ^∗∗^*p* > 0.01 relative to CL-GM. ^σ^*p* > 0.05, ^σσ^*p* > 0.01 relative to PL-GM.

Fourier transform Infrared analysis revealed that lipid content (*n* = 6 animals) was markedly reduced in the primary PS-GM (0.21 ± 0.010) when compared to the PL-GM (0.55 ± 0.028) and L-WM (0.89 ± 0.045) in the ipsilesional hemisphere. The amount of the lipid in PS-GM, PL-GM and L-WM were greatly reduced when compared with the contra-lesioned white matter (CL-WM) (1.1 ± 0.055) and contra-lesioned gray matter (CL-GM) (0.72 ± 0.036). These results were further compared with the healthy native control GM (0.79 ± 0.040) and WM (1.32 ± 0.066), where higher lipid content in comparison with the ipsilateral and contralateral hemisphere of the experimental animals (**Figures 3D,F** and see also **Figure 4L**). The results also revealed that the lipid acyl content (CH_2_) concentration in the PS-GM (0.264 ± 0.019), PL-GM (0.522 ± 0.026), and L-WM (0.563 ± 0.028) decreased compared to the contralateral region (0.6012 ± 0.030) of the ischemic brain, and all values were less than the healthy native animal (0.649 ± 0.035) as presented in (**Figures 4C,D,M**).

In converse, the degree of unsaturated components olefinic = CH increased, in the PL-GM (0.0049 ± 0.0002) and L-WM (0.0062 ± 0.0003), compared to the contralateral hemisphere (0.0026 ± 0.0002) and the healthy native animal (0.0014 ± 0.0001) as shown in **Figures 4E,F,N**. These results also indicated that the CH_3_ (methyl concentration) is severely reduced within the PS-GM (0.0048 ± 0.0002). On the other hand, the methyl concentration increased in the PL-GM (0.0494 ± 0.003) and L-WM (0.0627 ± 0.003) in the ipsilateral hemisphere in comparison to the contralateral hemisphere (0.0276 ± 0.0014) and the healthy native brain (0.0111 ± 0.0005) as represented in **Figures 4G,H,O**, respectively. Also documented was that the value of lipid ester (C = O) in the PS-GM region (0.11 ± 0.0055) was markedly reduced compared to PL-GM (0.64 ± 0.032) and L-WM (0.75 ± 0.0375) regions. Interestingly, lipid ester (C = O) amount in the contralateral hemisphere (0.28 ± 0.014) and native healthy brain (0.15 ± 0.0075) was lower than the PL-GM and L-WM as shown in **Figures 4I,J,P**.

The FTIR spectra revealed that total protein distribution in the cortex, especially in the PS-GM and PL-GM was greatly affected, due to stroke and, in the focal point of the stroke, there was protein aggregation. These analyses revealed that the protein level in the PS-GM (3.2 ± 0.16) was lower compared to PL-GM (12 ± 0.61). PS-GM and PL-GM experience severe reduction in protein in comparison to contralateral hemisphere (14.6 ± 0.73) and native healthy brain (17.8 ± 0.89) as presented in **Figures 3E,G** and see also **Figure 4K**. Moreover, the second derivative of the FTIR, range 1700–1600 cm^-1^, specifically protein band at the spectral range of 1630–1625 cm^-1^, indicates protein aggregation in the affected stroke region (**Figure 3J**), i.e., an increase in β-sheet and decrease in α-helix structures at 1660–1655 cm^-1^ as shown in the CL-GM and PS-GM spectra curve fitting (**Figures 3K,L**, respectively). The results indicate that the PS-GM (1.2 ± 0.06) region had increased aggregated protein content in comparison with the PL-GM (0.48 ± 0.024) and contralateral hemisphere (0.18 ± 0.009) and these two regions were having higher value than the native control (0.08 ± 0.004) (**Figure 3M**). (These findings correlate well with the histochemistry results see **Figure 2**). Here the results identified a decrease in the relative amount of lipid and protein bio-content in the PL-GM region of the ischemic brain in comparison to the native healthy control brain. Changes in the lipid/protein ratio (**Figures 4A,B**) suggest that these biochemical alterations associated with the stroke are due to triggering and initiating specific mechanisms of neurodegeneration.

The FTIR assays identified that the CC in the experimental animal (lesioned) brain, experienced reduction in lipid content (0.91 ± 0.0455) in comparison to the native healthy control animal (1.34 ± 0.067) (**Figures 3D,G**). The results also showed that that the lipid acyl (CH_2_) amounts in the lesioned CC (0.582 ± 0.0291) decreased compared to the native animal (0.661 ± 0.033) (**Figures 4C,D**). There is also an increase in the unsaturated components olefinic = CH (0.0058 ± 0.0003) compared to the healthy animal (0.0040 ± 0.0002) (**Figures 4E,F**). These results also indicated that the CH_3_ (methyl group) concentration in the lesioned CC (0.0574 ± 0.00278) increased compared to the native animal (0.0439 ± 0.0022) (**Figures 4G,H**). The lipid ester (C = O) concentration in the lesioned brain CC (0.64 ± 0.032) also experienced an increase in comparison to native animal (0.52 ± 0.026) (*p* = 0.73, *n* = 6) as shown in **Figures 4I,J**.

**FIGURE 5:**
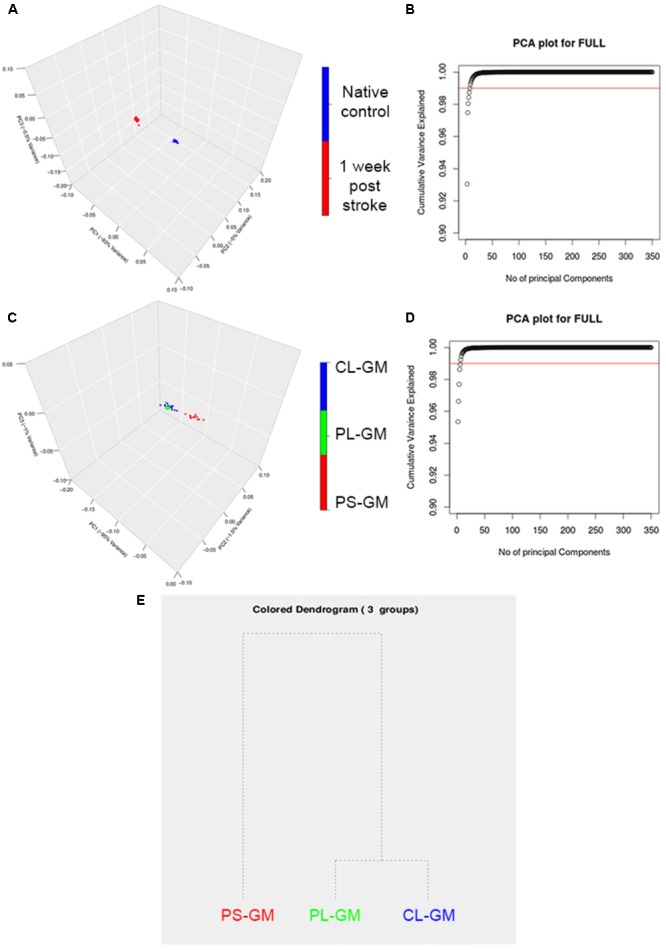
Principle component analysis (PCA) analyses of averaged FTIR spectroscopic studies. **(A)** Representative 3D score plot for PCA analysis based on the average FTIR spectral data in the region of 4000–700 cm^-1^ (with the range of 2500–2000 cm^-1^ removed) collected from the time point of native healthy control and stroke affected rat brain. **(B)**. The score plot of the PCA shows that there are three main PCs that separate the time point native control healthy and the affected stroke rat brains and are accounted for PC1 (93%), PC2 (4%), and PC3 (1%). **(C)** Represents the PCs plot that shows that there are the three main PCs that separate PS-GM, PL-GM, and CL-GM spectra. **(D)** The score plot of the PCA and shows that there are three main PCs that separate the PS-GM, PL-GM, and CL-GM spectra are counted for PC1 (95%), PC2 (1.5%), and PC3 (1%). **(E)** Represents the hierarchical dendrogram that shows a clear separation PS-GM, PL-GM, and CL-GM regions in the ipsilateral side of 1-week post ischemic stroke. The hierarchical dendrogram shows that PS-GM is located in one group while the PL-GM and CL-GM regions are located in the second group.

As a further analysis of the FTIR data, PCA was performed on the spectral second derivatives in the range of 4000–700 cm^-1^ (with the range of 2500–2000 cm^-1^ removed) for the native control and lesioned rat brain sections (**Figure 5A**). The differences were mainly significant in the region of 3994–2800 cm^-1^, which correlates to the lipid bio-molecular changes in the primary lesion. Also, there were molecular changes in the spectral region of 1530–1680 cm^-1^ which is related to protein structure in the tissue section. The scores plot revealed that the spectral data collected from the rat cortices were clustered into two distinct groups that correlate with healthy (native) and stroke affected. The clear segregation between the two groups in the PCA plots revealed that the molecular makeup of the cortical tissue has changed due to ischemic stroke with the first 3PCs accounting for ∼98.5% of the total variation as shown in **Figure 5B**. The PCA analysis also resulted in clustering of the PS-GM, PL-GM, and CL-GM (**Figure 5C**) from the scores plot of the three significant components: PC1 (95%), PC2 (1.5%), and PC3 (1%) as shown in **Figure 5D**. The first cluster contains the spectra from the PS-GM region, while the second group contains the spectra from PL-GM and CL-GM with this separation clearly shown in the hierarchical dendrogram (**Figure 5E**). The advantage of PCA is to allow the rat brain cortices to be clearly distinguished into healthy (control) and stroke region. Moreover, **Figure 5B** outlines an estimate that more than ∼98.5% of the variance in the spectra can be captured by the first three PCs. Furthermore, the PCA result makes it feasible to perform hierarchical clustering, thereby, combining PS-GM region with PL-GM and CL-GM regions using a dendrogram.

### Elemental Analysis by Laser Ablation (LA-ICPMS)

**FIGURE 6:**
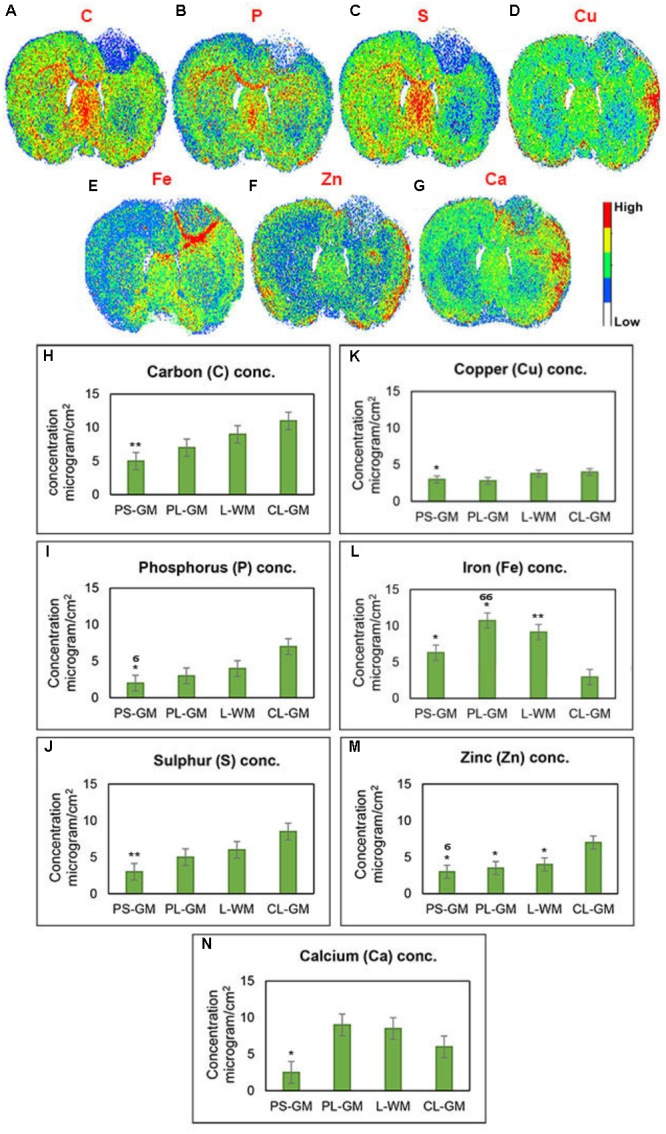
Qualitative elemental maps for whole rat brain sections. **(A–G)** Represent images of the elemental distributions of C, P, S, Cu, Fe, Zn, and Ca, respectively, in the rat brain section 1-week post-ischemic stroke measured by LA-ICP-QMS. **(H–N)** Elemental concentration of C, P, S, Cu, Fe, Zn, and Ca, respectively, from four regions of interest such as PS-GM, PL-GM, L-GM, and CL-GM. These LA images were color-labeled according to the calculated concentration values, where red corresponds to the highest concentration and white corresponds to the lowest concentration as shown on the color bars in the figures. Statistical significance was determined from six animals with a paired *t*-test and the 95% confidence interval. Bars represent mean ± SD. ^∗^*p* > 0.05, ^∗∗^*p* > 0.01 relative to CL-GM. ^σ^*p* > 0.05, ^σσ^*p* > 0.01 relative to PL-GM.

Employing LA-ICP-MS can be used to assess the elemental distributions of C, P, S, Cu, Fe, Zn, and Ca in the rat brain section 1-week post-stroke (**Figures 6A–G**). Here element values from the ipsilateral cortex (lesioned hemisphere) were compared with those of the contralateral cortex. The LA-ICPMS results provide three main observations: (a) a reduction in all element concentrations (Ca, C, Fe, Cu, S, P, and Zn) inside the stroke focus point (PS-GM) region; (b) Ca, Cu, and Zn were mainly accumulated at the edge of the injured brain hemisphere; and (c) significant and high accumulation of Fe around the PS-GM and in the lesion rim, shown in detail in **Figures 6H–N**. Further, the results indicate that the distribution of the brain elements were greatly affected by the ischemic stroke. This abnormal distribution might also effect brain functions involved in muscle control, movement and memory (Prashanth et al., 2015; Chen et al., 2016).

## Discussion

In this study diverse techniques were applied to delineate bio-chemical and elemental alterations 1-week post-stroke in the PS-GM, PL-GM, L-WM, CL-WM, CL-GM, and CC. A combination of FTIR and LA-ICPMS with PCA analysis, yielded novel results: significant changes in lipid values; protein conformation and in elemental distribution in multiple regions, which may correlate with morphological changes. For example, FTIR studies identified significantly enhanced protein aggregation, which included Aβ_1-42_ – one of the pathological markers of AD (Prashanth et al., 2015; Baldassarre et al., 2016; Chen et al., 2016). Moreover, we detected Aβ_1-42_ in the ipsilateral lesioned WM – further proof that the site of stroke is more global than originally thought. It should be noted that we do not detect Aβ_1-42_ aggregates in the contra-lateral WM indicative that the lesion induces APP/Aβ aggregation in the ipsilateral side. Furthermore, the breakdown of MBP and decrease in the myelin sheath thickness, post-ischemic stroke, which increases the vulnerability of exposed axons and leads to a decline in functional connectivity and behavioral deficits (Li et al., 2014; Baldassarre et al., 2016). Notably, other studies have reported that MBP dysfunction(s) were associated with physiological response to stress and emotional states, including anxiety and depression in adulthood, all possible indicators for commonalities in brain traumas (Drevets et al., 2008; Edgar and Sibille, 2012).

Ischemic stroke causes extensive brain damage through the generation of free radicals such as ROS O_2_^-^ (Olmez and Ozyurt, 2012) which attack polyunsaturated fatty acid (PUFA) containing structures (Allen and Bayraktutan, 2009). FTIR imaging identified a change in the lipid/protein ratio in the PL-GM region an event triggered by specific processes that lead to bio-chemical changes. The histological data revealed that the insulted brain experienced edema. Previous studies reported that edema initiates oxidative stress and increased protein oxidation and protein mis-folding and cell degeneration (Hackett et al., 2015a). Edema and oxidative stress may be the driving factors in lipid fragmentation, protein aggregation (mis-folding) (Hackett et al., 2015a), neurodegeneration (Klein and Ackerman, 2003; Uttara et al., 2009) and cell death (Ryter et al., 2007). Current bio-diagnostic techniques, such as MRI and biochemical assays are not sensitive enough to detect oxidative stress. Whereas, FTIR imaging spectroscopy is, to our knowledge, the only technique sensitive enough to detect these molecular changes associated with oxidative stress (Kneipp et al., 2000; Wang et al., 2005; Ozek et al., 2010; Caine et al., 2016). Thus, in order to understand the lipid alteration associated with ischemic stroke, FTIR spectra of lipids were analyzed. In the brain sections, we found significant alteration in the lipid acyl (CH_2_), lipid ester (C = O); olefinic = CH and methyl group (CH_3_). When double bond sites of PUFA are attacked by free radicals this leads to degradation of lipids into smaller fragments (Allen and Bayraktutan, 2009; Olmez and Ozyurt, 2012; Caine et al., 2016). Lipid peroxidation is associated with increased methyl (CH_3_) concentration and the formation of degradation products such as alkanes, carbonyl compounds, lipid aldehydes and alkyl radicals (Lamba et al., 1994; de Zwart et al., 1999; Manda et al., 2007; Yin et al., 2011).

Ischemic stroke caused reduction in the total lipid and lipid acyl contents at stroke focal PS-GM, PL-GM, and L-WM in the ipsilateral hemisphere. In PS-GM, total lipid decreased, as expected, due to tissue loss, evidenced also as protein loss. Hence, lipid acyl (CH_2_), lipid ester (C = O), olefinic = CH, and methyl (CH_3_) all declined significantly. However, in contrast to the PS-GM in the PL-GM, L-WM, and CC we observed a significant increase in lipid ester (C = O), olefinic = CH and methyl (CH_3_). An increase in the olefinic = CH content at the ipsilateral hemisphere compared with contralateral and native controls suggests that stroke induced an increase in the unsaturated fatty acids. Although, lipid peroxidation should result in loss of olefinic = CH bonds, but interestingly in our study we observed an increase in the olefinic = CH bio-content, which can be due to accumulation of double bond end products through lipid peroxidation compensation mechanisms (Liu et al., 2002; Severcan et al., 2005). Our results are in good agreement with previous studies on the effect of the lipid peroxidation on diabetic rat liver microsomal membranes and diabetic patients’ platelets (Liu et al., 2002; Severcan et al., 2005). These studies highlighted that lipid peroxidation induced an increase in the olefinic = CH content, which originated mainly from double bond lipid peroxidation products (Kar et al., 2004; Gouras et al., 2010). Our results also show an increase in the lipid ester (C = O) post stroke. Thus, our results emphasize that ischemic stroke induced lipid peroxidation which results in marked decreases in the total lipid and lipid CH_2_ contents associated with increases in the lipid ester (C = O), olefinic = CH and CH_3_ bio-contents (Lamba et al., 1994; de Zwart et al., 1999; Manda et al., 2007; Cakmak et al., 2011). These bio-chemical alterations occurs at the PL-GM, L-WM and the CC but were prominent at L-WM and the CC due to higher lipid content.

From immunohistochemical studies we noted high GFAP expression, an indicator for astrogliosis, and from elemental data enhanced Fe levels-a possible marker for increased mitochondria; in all, a likelihood that glia are metabolizing lipids esters and olefins, including removing necrotic tissue. In the WM, below the site of the lesion, we again noted a decrease in total lipid content, which was associated with significant increase in lipid ester, olefin, and CH_3_ indicative that possibly degradation of long chain fats was occurring, including cell membranes. Intriguingly at this locus, we detected only Aβ_1-42_, which is thought to be formed due to exposure to reactive oxygen species.

Protein exposure to reactive oxygen results in protein cross-linking, aggregation, fragmentation, and denaturation, resulting in loss of function. The levels of the total protein and aggregated protein were calculated from the amide I band at 1700–1600 cm^-1^ and curve fitting at 1630 cm^-1^, respectively. As expected from histological data the FTIR amide I data indicated a loss of protein from the lesioned GM (PS-GM) compared with the control samples. Interestingly, we observed that there is an increase in the band absorption of β-sheets at 1630 cm^-1^ and associated decrease of α- helical protein band absorption at 1655 cm^-1^ indicating protein aggregation. This protein conformational change was not detected on the contra-lateral side, which indicate that stroke induces protein alterations. The PS-GM and PL-GM regions experienced a decrease in total protein levels (amide values), with the most surprising and dramatic change in aggregated protein in these areas. Histochemical studies identified significant changes in APP in the infarcted area (closely associated with glial cells) and tests for Aβ_1-42_- which has been previously reported to increase, not only in stroke, but also in other neurodegenerative disorders (Paul Murphy and LeVine, 2010; Esparza et al., 2016; Wildburger et al., 2017). It should be noted no Tau protein was detected in the lesioned area, thus it was inferred that the protein aggregation was exclusively APP derived and was Aβ_1-42_. Moreover, it is tempting to speculate that Aβ_1-42_ accumulation due to ischemic stroke might also trigger other neurological deficits and subsequent neurodegeneration (Kar et al., 2004; Gouras et al., 2010).

Elemental studies clearly defined significant loss of C, P, and S from the PS-GM, PL-GM, and the L-WM as these are the major components of nucleic acids, protein and lipids that make up cells. Sulfur concentration were greatly reduced in the ipsilateral hemisphere. Sulfur is a constituent of the anti-oxidant enzymes glutathione peroxidases (GP) and thioredoxin reductases (TR) and of the metabolic enzyme *S*-adenosylhomocysteine (S-ACH) (Zima et al., 1996; Birben et al., 2012; Li et al., 2013). In several neurodegenerative disorders loss of these enzymes has been postulated as a corollary of enhanced cell pathology (Zima et al., 1996; Birben et al., 2012; Li et al., 2013). Surprisingly, in the cortices we identified significantly high levels of Ca and Fe. From studies in the hippocampus, high Ca^2+^ levels were found to be excitotoxic (Dong et al., 2009) and in the brain Fe ions can increase the formation of reactive radicals and thus lipid peroxidation (Halliwell and Gutteridge, 1986). This change can also be attributed to an increase in to astroglial cells that metabolizing necrotic tissue. In our study, a small change in Cu values was found, but this could be linked to the fact that Cu and Zn are bound to synaptic termini and regulate exocytosis (Sadiq et al., 2012), thus the possibility is that release of these metals into the micro-environment may account for the increased neuronal excitability that has been reported by others after stroke (Li and Zhang, 2012). Our results indicate that ischemic stroke can induce significant changes in the stroke focus point, the GM, as well as in the adjacent WM of the affected hemisphere. Furthermore, the enriched lipid brain regions, especially brain WM are vulnerable to ischemic stroke and can be highly affected by the production of free radicals and bi-products of oxidation.

The chemometric analyses, PCA in the spectral range of 3500–900 cm^-1^ and the hierarchy dendrogram from HCA, delineated a clear separation between the control and the lesioned sections. From the PCA plot, it was clear that the primary stroke PS-GM clustered in one group while both PL-GM and CL-GM clustered together. This clear separation reveals that the stroke focus region PS-GM has significant molecular changes in its bio-chemical makeup. One of these major bio-molecular alterations was found by curve fitting of the second derivative of the protein bands in the PS-GM spectrum. It revealed an increase in the β-sheet content and an associated decrease in α- helical secondary structure- again the interpretation being that protein aggregation, a marker for neurodegeneration, occurred. The data also indicate that the effects of stroke are much more global on the ipsilateral side than originally thought. Thus, newer or more improved ischemic stroke management in the sub-acute phase should prioritize treating edema and the oxidative stress damage in order to have a successful outcome to maintain and improve the PL-GM and L-WM brain tissue.

Our findings suggest that edema and oxidative stress are the major factors that affect the integrity, structure and functionality of the brain as well as leading to bio-molecular and elemental alterations 1 week post-stroke. However, it has been found that 24 h post stroke, the molecular and elemental alterations are mainly due to tissue swelling following edema but oxidative-damage is not responsible for the these alterations especially at perilesional gray matter (PL-GM) region (Caine et al., 2016). Our results show that FTIR bio-spectroscopy is a non-destructive, rapid, and a refined technique to characterize oxidative stress bio-markers not obtained by routine approaches for lipid degradation. Also, we can detect secondary structure protein changes such as β-sheet formation. Normally, detection of these changes requires biochemical methods that include tissue homogenization and, or treatment for immunoblotting or IHC, destroying the spatial-temporal integrity of the brain. In summary, these results suggest that a good therapeutic strategy should include a mechanism that provides protective effect from brain swelling (edema) and neurotoxicity by scavenging the lipid peroxidation end products. This strategy can include the protection of the blood–brain barrier (BBB) endothelial cells. Thus, we conclude that FTIR can complement and expedite research into stroke.

## Author Contributions

MA and FR performed all the FTIR and LA measurements in this study. MA, TA, KA-S, and EA were involved in data interpretation, drafting and editing the manuscript. AL was involved in elemental analysis studies. EA and NM were involved in IHC studies. RM and EU were involved in processing the experimental data and performing the multivariate and statistical analysis. All authors discussed the results and commented on the manuscript and in critical revision of this manuscript.

## Conflict of Interest Statement

The authors declare that the research was conducted in the absence of any commercial or financial relationships that could be construed as a potential conflict of interest. The reviewer AE declared a shared affiliation, with no collaboration, with several of the authors, FR and KA-S, to the handling Editor at the time of the review.

## Supplementary Material

FIGURE S1 The representative FTIR spectrum of white matter (WM) in a native control rat brain in the spectral range of 4000–700 cm^-1^ and mechanical properties of the native and lesioned brain sections. Representative FTIR spectra of WM in the native control rat brain from region 4000–700 cm^-1^.

TABLE S1 Fourier transform Infrared (FTIR) spectral bands and their corresponding bio-chemical assignment.

## Abbreviations

CC: corpus callosum
CL-GM: contra-lesioned gray matter
CL-WM: contra-lesioned white matter
FTIR: Fourier transform Infrared
GM: gray matter
IHC: immunohistochemistry
LA-ICPMS: laser-ablation inductively-coupled-plasma mass-spectrometry
L-WM: lesioned white matter
PL-GM: perilesioned gray matter
PS-GM: primary stroke lesion gray matter
ROI: regions of interest
TC-GM: time control gray matter
WM: white matter

## References

[B1] Ali M. H. Al-Saad K. Popelka A. van Tilborg G. Goormaghtigh E. (2016). Application of Fourier transform infrared (FTIR) spectroscopy and atomic force microscopy in stroke-affected brain tissue. *Swift J. Med. Med. Sci.* 2 011–024.

[B2] Allen C. L. Bayraktutan U. (2009). Oxidative stress and its role in the pathogenesis of ischaemic stroke. *Int. J. Stroke* 4 461–470. 10.1111/j.1747-4949.2009.00387.x 19930058

[B3] Ami D. Mereghetti P. Doglia S. M. (2013). “Multivariate analysis for Fourier transform infrared spectra of complex biological systems and processes,” in *Multivariate Analysis in Management, Engineering and the Sciences* ed. Akselsen B. (London: InTech).

[B4] Baker M. J. Trevisan J. Bassan P. Bhargava R. Butler H. J. Dorling K. M. (2014). Using Fourier transform IR spectroscopy to analyze biological materials. *Nat. Protoc.* 9 1771–1791. 10.1038/nprot.2014.110 24992094 PMC4480339

[B5] Balbekova A. Lohninger H. van Tilborg G. A. Dijkhuizen R. M. Bonta M. Limbeck A. (2017). Fourier transform infrared (FT-IR) and laser ablation inductively coupled plasma–mass spectrometry (LA-ICP-MS) imaging of cerebral ischemia: combined analysis of rat brain thin cuts toward improved tissue classification. *Appl. Spectrosc.* 72 241–250. 10.1177/0003702817734618 28905634

[B6] Baldassarre A. Ramsey L. E. Siegel J. S. Shulman G. L. Corbetta M. (2016). Brain connectivity and neurological disorders after stroke. *Curr. Opin. Neurol.* 29 706–713. 10.1097/WCO.0000000000000396 27749394 PMC5682022

[B7] Bassan P. Kohler A. Martens H. Lee J. Byrne H. J. Dumas P. (2010). “Resonant MIE scattering (RMieS) correction of infrared spectra from highly scattering biological samples”. *Analyst* 135 268–277. 10.1039/b921056c 20098758

[B8] Becker J. S. Matusch A. Becker J. S. Wu B. Palm C. Becker A. J. (2011). Mass spectrometric imaging (MSI) of metals using advanced BrainMet techniques for biomedical research. *Int. J. Mass Spectrom.* 307 3–15. 10.1016/j.ijms.2011.01.015

[B9] Becker J. S. Matusch A. Wu B. (2014). Bioimaging mass spectrometry of trace elements–recent advance and applications of LA-ICP-MS: a review. *Anal. Chim. Acta* 835 1–18. 10.1016/j.aca.2014.04.048 24952624

[B10] Becker R. A. Chambers J. M. Wilks A. R. (1988). *The New S Language.* Monterey, CA: Wadsworth & Brooks/Cole.

[B11] Benseny-Cases N. Klementieva O. Cotte M. Ferrer I. Cladera J. (2014). Microspectroscopy (μFTIR) reveals co-localization of lipid oxidation and amyloid plaques in human Alzheimer disease brains. *Anal. Chem.* 86 12047–12054. 10.1021/ac502667b 25415602

[B12] Birben E. Sahiner U. M. Sackesen C. Erzurum S. Kalayci O. (2012). Oxidative stress and antioxidant defense. *World Allergy Organ. J.* 5 9–19. 10.1097/WOX.0b013e3182439613 23268465 PMC3488923

[B13] Bretón R. R. Rodríguez J. C. G. (2012). “Excitotoxicity and oxidative stress in acute ischemic stroke,” in *Acute Ischemic Stroke* ed. Rodríguez J. C. G. (London: InTech).

[B14] Broughton B. R. Reutens D. C. Sobey C. G. (2009). Apoptotic mechanisms after cerebral ischemia. *Stroke* 40 e331–e339. 10.1161/STROKEAHA.108.531632 19182083

[B15] Brouns R. De Vil B. Cras P. De Surgeloose D. Mariën P. De Deyn P. P. (2010). Neurobiochemical markers of brain damage in cerebrospinal fluid of acute ischemic stroke patients. *Clin. Chem.* 56 451–458. 10.1373/clinchem.2009.134122 19959621

[B16] Caine S. Hackett M. J. Hou H. Kumar S. Maley J. Ivanishvili Z. (2016). A novel multi-modal platform to image molecular and elemental alterations in ischemic stroke. *Neurobiol. Dis.* 91 132–142. 10.1016/j.nbd.2016.03.006 26969531

[B17] Cakmak G. Miller L. M. Zorlu F. Severcan F. (2012). Amifostine, a radioprotectant agent, protects rat brain tissue lipids against ionizing radiation induced damage: an FTIR microspectroscopic imaging study. *Arch. Biochem. Biophys.* 520 67–73. 10.1016/j.abb.2012.02.012 22402174

[B18] Cakmak G. Zorlu F. Severcan M. Severcan F. (2011). Screening of protective effect of amifostine on radiation-induced structural and functional variations in rat liver microsomal membranes by FT-IR spectroscopy. *Anal. Chem.* 83 2438–2444. 10.1021/ac102043p 21410135

[B19] Carmichael S. T. (2003). Plasticity of cortical projections after stroke. *Neuroscientist* 9 64–75. 10.1177/1073858402239592 12580341

[B20] Carter E. A. Rayner B. S. McLeod A. I. Wu L. E. Marshall C. P. Levina A. (2010). Silicon nitride as a versatile growth substrate for microspectroscopic imaging and mapping of individual cells. *Mol. Biosyst.* 6 1316–1322. 10.1039/c001499k 20445927

[B21] Cheatwood J. L. Emerick A. J. Kartje G. L. (2008). Neuronal plasticity and functional recovery after ischemic stroke. *Top. Stroke Rehabil.* 15 42–50. 10.1310/tsr1501-42 18250073

[B22] Chen P. Miah M. R. Aschner M. (2016). Metals and neurodegeneration. *F1000Res.* 5:F1000FacultyRev-366. 10.12688/f1000research.7431.1 27006759 PMC4798150

[B23] de Zwart L. L. Meerman J. H. Commandeur J. N. Vermeulen N. P. (1999). Biomarkers of free radical damage: applications in experimental animals and in humans. *Free Radic. Biol. Med.* 26 202–226. 10.1016/S0891-5849(98)00196-8 9890655

[B24] Deb P. Sharma S. Hassan K. M. (2010). Pathophysiologic mechanisms of acute ischemic stroke: an overview with emphasis on therapeutic significance beyond thrombolysis. *Pathophysiology* 17 197–218. 10.1016/j.pathophys.2009.12.001 20074922

[B25] Dietrich W. D. Ginsberg M. D. Busto R. Watson B. D. (1986). Photochemically induced cortical infarction in the rat. Acute and subacute alterations in local glucose utilization. *J. Cereb. Blood Flow Metab.* 6 195–202. 10.1038/jcbfm.1986.32 3958064

[B26] Dirnagl U. Iadecola C. Moskowitz M. A. (1999). Pathobiology of ischaemic stroke: an integrated view. *Trends Neurosci.* 22 391–397. 10.1016/S0166-2236(99)01401-0 10441299

[B27] Dong X. X. Wang Y. Qin Z. H. (2009). Molecular mechanisms of excitotoxicity and their relevance to pathogenesis of neurodegenerative diseases. *Acta Pharmacol. Sin.* 30 379–387. 10.1038/aps.2009.24 19343058 PMC4002277

[B28] Dostovic Z. Dostovic E. Smajlovic D. Ibrahimagic O. C. Avdic L. (2016). Brain edema after ischaemic stroke. *Med. Arch.* 70 339–341. 10.5455/medarh.2016.70.339-341 27994292 PMC5136437

[B29] Drevets W. C. Price J. L. Furey M. L. (2008). Brain structural and functional abnormalities in mood disorders: implications for neurocircuitry models of depression. *Brain Struct. Funct.* 213 93–118. 10.1007/s00429-008-0189-x 18704495 PMC2522333

[B30] Edgar N. Sibille E. (2012). A putative functional role for oligodendrocytes in mood regulation. *Transl. Psychiatry* 2:e109. 10.1038/tp.2012.34 22832953 PMC3365253

[B31] Esparza T. J. Wildburger N. C. Jiang H. Gangolli M. Cairns N. J. Bateman R. J. (2016). Soluble amyloid-beta aggregates from human Alzheimer’s disease brains. *Sci. Rep.* 6:38187. 10.1038/srep38187 27917876 PMC5137165

[B32] Garcia J. H. (1984). Experimental ischemic stroke: a review. *Stroke* 15 5–14. 10.1161/01.STR.15.1.5 6364464

[B33] Goormaghtigh E. (2016). Infrared imaging in histopathology: is a unified approach possible? *Biomed. Spectrosc. Imaging* 5 325–346. 10.3233/BSI-160151

[B34] Gouras G. K. Tampellini D. Takahashi R. H. Capetillo-Zarate E. (2010). Intraneuronal β-amyloid accumulation and synapse pathology in Alzheimer’s disease. *Acta Neuropathol.* 119 523–541. 10.1007/s00401-010-0679-9 20354705 PMC3183823

[B35] Hackett M. J. DeSouza M. Caine S. Bewer B. Nichol H. Paterson P. G. (2015a). A new method to image heme-Fe, total Fe, and aggregated protein levels after intracerebral hemorrhage. *ACS Chem. Neurosci.* 6 761–770. 10.1021/acschemneuro.5b00037 25695130 PMC4490904

[B36] Hackett M. J. Lee J. El-Assaad F. McQuillan J. A. Carter E. A. Grau G. E. (2015b). A new method to image heme-Fe, total Fe, and aggregated protein levels after intracerebral hemorrhage. *ACS Chem. Neurosci.* 6 761–770. 10.1021/acschemneuro.5b00037 25695130 PMC4490904

[B37] Halliwell B. Gutteridge J. M. (1986). Iron and free radical reactions: two aspects of antioxidant protection. *Trends Biochem. Sci.* 11 372–375. 10.1016/0968-0004(86)90207-0

[B38] Hare D. Reedy B. Grimm R. Wilkins S. Volitakis I. George J. L. (2009). Quantitative elemental bio-imaging of Mn, Fe, Cu and Zn in 6-hydroxydopamine induced Parkinsonism mouse models. *Metallomics* 1 53–58. 10.1039/c0mt00039f 21072366

[B39] Hare D. J. George J. L. Grimm R. Wilkins S. Adlard P. A. Cherny R. A. (2010). Three-dimensional elemental bio-imaging of Fe, Zn, Cu, Mn and P in a 6-hydroxydopamine lesioned mouse brain. *Metallomics* 2 745–753. 10.1039/c0mt00039f 21072366

[B40] Harrison J. P. Berry D. (2017). Vibrational spectroscopy for imaging single microbial cells in complex biological samples. *Front. Microbiol.* 8:675. 10.3389/fmicb.2017.00675 28450860 PMC5390015

[B41] Heraud P. Caine S. Campanale N. Karnezis T. McNaughton D. Wood B. R. (2010). Early detection of the chemical changes occurring during the induction and prevention of autoimmune-mediated demyelination detected by FT-IR imaging. *Neuroimage* 49 1180–1189. 10.1016/j.neuroimage.2009.09.053 19796690

[B42] Hu X. De Silva T. M. Chen J. Faraci F. M. (2017). Cerebral vascular disease and neurovascular injury in ischemic stroke. *Circ. Res.* 120 449–471. 10.1161/CIRCRESAHA.116.308427 28154097 PMC5313039

[B43] Huang L. Wu Z. B. ZhuGe Q. Zheng W. Shao B. Wang B. (2014). Glial scar formation occurs in the human brain after ischemic stroke. *Int. J. Med. Sci.* 11 344–348. 10.7150/ijms.8140 24578611 PMC3936028

[B44] Kar S. Slowikowski S. P. Westaway D. Mount H. T. (2004). Interactions between β-amyloid and central cholinergic neurons: implications for Alzheimer’s disease. *J. Psychiatry Neurosci.* 29 427–441. 15644984 PMC524960

[B45] Kastyak M. Z. Szczerbowska-Boruchowska M. Adamek D. Tomik B. Lankosz M. Gough K. M. (2010). Pigmented creatine deposits in Amyotrophic Lateral Sclerosis central nervous system tissues identified by synchrotron Fourier Transform Infrared microspectroscopy and X-ray fluorescence spectromicroscopy. *Neuroscience* 166 1119–1128. 10.1016/j.neuroscience.2010.01.017 20097271

[B46] Kazarian S. G. Chan K. L. A. (2006). Applications of ATR-FTIR spectroscopic imaging to biomedical samples. *Biochim. Biophys. Acta* 1758 858–867. 10.1016/j.bbamem.2006.02.011 16566893

[B47] Klein J. A. Ackerman S. L. (2003). Oxidative stress, cell cycle, and neurodegeneration. *J. Clin. Invest.* 111 785–793. 10.1172/JCI200318182 12639981 PMC153779

[B48] Kneipp J. Lasch P. Baldauf E. Beekes M. Naumann D. (2000). Detection of pathological molecular alterations in scrapie-infected hamster brain by Fourier transform infrared (FT-IR) spectroscopy. *Biochim. Biophys. Acta* 1501 189–199. 10.1016/S0925-4439(00)00021-1 10838192

[B49] Kohler A. Böcker U. Shapaval V. Forsmark A. Andersson M. Warringer J. (2015). High-throughput biochemical fingerprinting of *Saccharomyces cerevisiae* by Fourier transform infrared spectroscopy. *PLoS One* 10:e0118052. 10.1371/journal.pone.0118052 25706524 PMC4338198

[B50] Kumar S. Shabi T. S. Goormaghtigh E. (2014). A FTIR imaging characterization of fibroblasts stimulated by various breast cancer cell lines. *PLoS One* 9:e111137. 10.1371/journal.pone.0111137 25390361 PMC4229076

[B51] Kuroiwa T. Xi G. Hua Y. Nagaraja T. N. Fenstermacher J. D. Keep R. F. (2009). Development of a rat model of photothrombotic ischemia and infarction within the caudoputamen. *Stroke* 40 248–253. 10.1161/STROKEAHA.108.527853 19038913 PMC2692300

[B52] Lai T. W. Zhang S. Wang Y. T. (2014). Excitotoxicity and stroke: identifying novel targets for neuroprotection. *Prog. Neurobiol.* 115 157–188. 10.1016/j.pneurobio.2013.11.006 24361499

[B53] Lamba O. P. Borchman D. Garner W. H. (1994). Spectral characterization of lipid peroxidation in rabbit lens membranes induced by hydrogen peroxide in the presence of Fe2^+^ Fe3^+^ cations: a site-specific catalyzed oxidation. *Free Radic. Biol. Med.* 16 591–601. 10.1016/0891-5849(94)90059-0 8026802

[B54] Lasch P. Boese M. Pacifico A. Diem M. (2002). FT-IR spectroscopic investigations of single cells on the subcellular level. *Vib. Spectrosc.* 28 147–157. 10.1016/S0924-2031(01)00153-9

[B55] Lee J. M. Grabb M. C. Zipfel G. J. Choi D. W. (2000). Brain tissue responses to ischemia. *J. Clin. Invest.* 106 723–731. 10.1172/JCI11003 10995780 PMC381398

[B56] Lekander I. Willers C. Von Euler M. Lilja M. Sunnerhagen K. S. Pessah-Rasmussen H. (2017). Relationship between functional disability and costs one and two years post stroke. *PLoS One* 12:e0174861. 10.1371/journal.pone.0174861 28384164 PMC5383241

[B57] Leskovjan A. C. Kretlow A. Miller L. M. (2010). Fourier transform infrared imaging showing reduced unsaturated lipid content in the hippocampus of a mouse model of Alzheimer’s disease. *Anal. Chem.* 82 2711–2716. 10.1021/ac1002728 20187625 PMC2848295

[B58] Li J. Li W. Jiang Z. G. Ghanbari H. A. (2013). Oxidative stress and neurodegenerative disorders. *Int. J. Mol. Sci.* 14 24438–24475. 10.3390/ijms141224438 24351827 PMC3876121

[B59] Li W. Li Y. Zhu W. Chen X. (2014). Changes in brain functional network connectivity after stroke. *Neural Regen. Res.* 9 51–60. 10.4103/1673-5374.125330 25206743 PMC4146323

[B60] Li Y. I. Chen J. Zhang C. L. Wang L. Lu D. Katakowski M. (2005). Gliosis and brain remodeling after treatment of stroke in rats with marrow stromal cells. *Glia* 49 407–417. 10.1002/glia.20126 15540231

[B61] Li Y. V. Zhang J. H. (eds) (2012). “Metal ions in stroke pathophysiology,” in *Metal Ion in Stroke* (New York, NY: Springer) 1–12. 10.1007/978-1-4419-9663-3_1

[B62] Liang D. Bhatta S. Gerzanich V. Simard J. M. (2007). Cytotoxic edema: mechanisms of pathological cell swelling. *Neurosurg. Focus* 22 1–9. 10.3171/foc.2007.22.5.3 PMC2740913 17613233

[B63] Liao C. R. Rak M. Lund J. Unger M. Platt E. Albensi B. C. (2013). Synchrotron FTIR reveals lipid around and within amyloid plaques in transgenic mice and Alzheimer’s disease brain. *Analyst* 138 3991–3997. 10.1039/c3an00295k 23586070

[B64] Liu K. Z. Bose R. Mantsch H. H. (2002). Infrared spectroscopic study of diabetic platelets. *Vib. Spectrosc.* 28 131–136. 10.1016/S0924-2031(01)00163-1

[B65] Lo E. H. Dalkara T. Moskowitz M. A. (2003). Neurological diseases: mechanisms, challenges and opportunities in stroke. *Nat. Rev. Neurosci.* 4 399–414. 10.1016/j.smim.2016.03.015 12728267

[B66] Manda K. Ueno M. Moritake T. Anzai K. (2007). α-Lipoic acid attenuates x-irradiation-induced oxidative stress in mice. *Cell Biol. Toxicol.* 23 129–137. 10.1007/s10565-006-0137-6 17094020

[B67] Matusch A. Becker J. S. (2012). Bio-imaging of metals in a mouse model of Alzheimer’s disease by laser ablation inductively coupled plasma mass spectrometry. *Biomed. Spectrosc. Imaging* 1 57–65.

[B68] Matusch A. Depboylu C. Palm C. Wu B. Höglinger G. U. Schäfer M. K. H. (2010). Cerebral bioimaging of Cu, Fe, Zn, and Mn in the MPTP mouse model of Parkinson’s disease using laser ablation inductively coupled plasma mass spectrometry (LA-ICP-MS). *J. Am. Soc. Mass Spectrom.* 21 161–171. 10.1016/j.jasms.2009.09.022 19892565

[B69] Matusch A. Fenn L. S. Depboylu C. Klietz M. Strohmer S. McLean J. A. (2012). Combined elemental and biomolecular mass spectrometry imaging for probing the inventory of tissue at a micrometer scale. *Anal. Chem.* 84 3170–3178. 10.1021/ac203112c 22413784 PMC5100675

[B70] Michinaga S. Koyama Y. (2015). Pathogenesis of brain edema and investigation into anti-edema drugs. *Int. J. Mol. Sci.* 16 9949–9975. 10.3390/ijms16059949 25941935 PMC4463627

[B71] Miller L. M. Bourassa M. W. Smith R. J. (2013). FTIR spectroscopic imaging of protein aggregation in living cells. *Biochim. Biophys. Acta* 1828 2339–2346. 10.1016/j.bbamem.2013.01.014 23357359 PMC3722250

[B72] Miller L. M. Wang Q. Telivala T. P. Smith R. J. Lanzirotti A. Miklossy J. (2006). Synchrotron-based infrared and X-ray imaging shows focalized accumulation of Cu and Zn co-localized with β-amyloid deposits in Alzheimer’s disease. *J. Struct. Biol.* 155 30–37. 10.1016/j.jsb.2005.09.004 16325427

[B73] Murphy T. H. Corbett D. (2009). Plasticity during stroke recovery: from synapse to behaviour. *Nat. Rev. Neurosci.* 10 861–872. 10.1038/nrn2735 19888284

[B74] Olmez I. Ozyurt H. (2012). Reactive oxygen species and ischemic cerebrovascular disease. *Neurochem. Int.* 60 208–212. 10.1016/j.neuint.2011.11.009 22122807

[B75] Ozek N. S. Tuna S. Erson-Bensan A. E. Severcan F. (2010). Characterization of microRNA-125b expression in MCF7 breast cancer cells by ATR-FTIR spectroscopy. *Analyst* 135 3094–3102. 10.1039/c0an00543f 20978686

[B76] Paul Murphy M. LeVine H. I. I. I. (2010). Alzheimer’s disease and the β-amyloid peptide. *J. Alzheimers Dis.* 19 311–323. 10.3233/JAD-2010-1221 20061647 PMC2813509

[B77] Petibois C. Desbat B. (2010). Clinical application of FTIR imaging: new reasons for hope. *Trends Biotechnol.* 28 495–500. 10.1016/j.tibtech.2010.07.003 20828847

[B78] Petibois C. Wehbe K. Belbachir K. Noreen R. Déléris G. (2009). Current trends in the development of FTIR imaging for the quantitative analysis of biological samples. *Acta Phys. Pol. A Gen. Phys.* 115:507 10.12693/APhysPolA.115.507

[B79] Pevsner P. H. Eichenbaum J. W. Miller D. C. Pivawer G. Eichenbaum K. D. Stern A. (2001). A photothrombotic model of small early ischemic infarcts in the rat brain with histologic and MRI correlation. *J. Pharmacol. Toxicol. Methods* 45 227–233. 10.1016/S1056-8719(01)00153-8 11755387

[B80] Prashanth L. Kattapagari K. K. Chitturi R. T. Baddam V. R. R. Prasad L. K. (2015). A review on role of essential trace elements in health and disease. *J. NTR Univ. Health Sci.* 4 75–85. 10.4103/2277-8632.158577

[B81] Prentice H. Modi J. P. Wu J. Y. (2015). Mechanisms of neuronal protection against excitotoxicity, endoplasmic reticulum stress, and mitochondrial dysfunction in stroke and neurodegenerative diseases. *Oxid. Med. Cell. Longev.* 2015:964518. 10.1155/2015/964518 26576229 PMC4630664

[B82] Pribic R. (1994). Principal component analysis of Fourier transform infrared and/or circular dichroism spectra of proteins applied in a calibration of protein secondary structure. *Anal. Biochem.* 223 26–34. 10.1006/abio.1994.1541 7695098

[B83] Ramasubbu R. Robinson R. G. Flint A. J. Kosier T. Price T. R. (1998). Functional impairment associated with acute post stroke depression: the Stroke Data Bank Study. *J. Neuropsychiatry Clin. Neurosci.* 10 26–33. 10.1176/jnp.10.1.26 9547463

[B84] Ryter S. W. Kim H. P. Hoetzel A. Park J. W. Nakahira K. Wang X. (2007). Mechanisms of cell death in oxidative stress. *Antioxid. Redox Signal.* 9 49–89. 10.1089/ars.2007.9.49 17115887

[B85] Sadiq S. Ghazala Z. Chowdhury A. Büsselberg D. (2012). Metal toxicity at the synapse: presynaptic, postsynaptic, and long-term effects. *J. Toxicol.* 2012:132671. 10.1155/2012/132671 22287959 PMC3263637

[B86] Sanchez G. (2011). *Visualizing Dendrograms in R.* Available at: http://rpubs.com/gaston/dendrograms

[B87] Sarti C. Rastenyte D. Cepaitis Z. Tuomilehto J. (2000). International trends in mortality from stroke, 1968 to 1994. *Stroke* 31 1588–1601. 10.1161/01.STR.31.7.1588 10884459

[B88] Severcan F. Gorgulu G. Gorgulu S. T. Guray T. (2005). Rapid monitoring of diabetes-induced lipid peroxidation by Fourier transform infrared spectroscopy: evidence from rat liver microsomal membranes. *Anal. Biochem.* 339 36–40. 10.1016/j.ab.2005.01.011 15766707

[B89] Shichita T. Ito M. Yoshimura A. (2014). Post-ischemic inflammation regulates neural damage and protection. *Front. Cell. Neurosci.* 8:319. 10.3389/fncel.2014.00319 25352781 PMC4196547

[B90] Simard J. M. Kent T. A. Chen M. Tarasov K. V. Gerzanich V. (2007). Brain oedema in focal ischaemia: molecular pathophysiology and theoretical implications. *Lancet Neurol.* 6 258–268. 10.1016/S1474-4422(07)70055-8 17303532 PMC2725365

[B91] Snögren M. Sunnerhagen K. S. (2009). Description of functional disability among younger stroke patients: exploration of activity and participation and environmental factors. *Int. J. Rehabil. Res.* 32 124–131. 10.1097/MRR.0b013e328325a5be 19339894

[B92] Srinivasan G. (2010). *Vibrational Spectroscopic Imaging for Biomedical Applications.* New York, NY: McGraw Hill Professional.

[B93] Szczerbowska-Boruchowska M. Dumas P. Kastyak M. Z. Chwiej J. Lankosz M. Adamek D. (2007). Biomolecular investigation of human *Substantia nigra* in Parkinson’s disease by synchrotron radiation Fourier transform infrared microspectroscopy. *Arch. Biochem. Biophys.* 459 241–248. 10.1016/j.abb.2006.12.027 17274943

[B94] Turker S. (2012). Application of infrared spectroscopy in the study of neurological diseases. *Biomed. Spectrosc. Imaging* 1 303–323.

[B95] Turker S. Severcan M. Ilbay G. Severcan F. (2014). Epileptic seizures induce structural and functional alterations on brain tissue membranes. *Biochim. Biophys. Acta* 1838 3088–3096. 10.1016/j.bbamem.2014.08.025 25194682

[B96] Uttara B. Singh A. V. Zamboni P. Mahajan R. T. (2009). Oxidative stress and neurodegenerative diseases: a review of upstream and downstream antioxidant therapeutic options. *Curr. Neuropharmacol.* 7 65–74. 10.2174/157015909787602823 19721819 PMC2724665

[B97] Van Bruggen N. Cullen B. M. King M. D. Doran M. Williams S. R. Gadian D. G. (1992). T2-and diffusion-weighted magnetic resonance imaging of a focal ischemic lesion in rat brain. *Stroke* 23 576–582. 10.1161/01.STR.23.4.576 1373254

[B98] Wang Q. Kretlow A. Beekes M. Naumann D. Miller L. (2005). In situ characterization of prion protein structure and metal accumulation in scrapie-infected cells by synchrotron infrared and X-ray imaging. *Vib. Spectrosc.* 38 61–69.

[B99] Wildburger N. C. Esparza T. J. LeDuc R. D. Fellers R. T. Thomas P. M. Cairns N. J. (2017). Diversity of amyloid-beta proteoforms in the Alzheimer’s disease brain. *Sci. Rep.* 7:9520. 10.1038/s41598-017-10422-x 28842697 PMC5572664

[B100] Xing C. Arai K. Lo E. H. Hommel M. (2012). Pathophysiologic cascades in ischemic stroke. *Int. J. Stroke* 7 378–385. 10.1111/j.1747-4949.2012.00839.x 22712739 PMC3985770

[B101] Yamamoto M. Shima T. Uozumi T. Sogabe T. Yamada K. Kawasaki T. (1983). A possible role of lipid peroxidation in cellular damages caused by cerebral ischemia and the protective effect of alpha-tocopherol administration. *Stroke* 14 977–982. 10.1161/01.STR.14.6.977 6659003

[B102] Yildirim A. Kotan D. Yildirim S. Ayguel R. Akçay F. (2007). Increased lipid peroxidation and decreased antioxidant response in serum and cerebrospinal fluid in acute ischemic stroke. *Turk. J. Med. Sci.* 37 75–81.

[B103] Yin H. Xu L. Porter N. A. (2011). Free radical lipid peroxidation: mechanisms and analysis. *Chem. Rev.* 111 5944–5972. 10.1021/cr200084z 21861450

[B104] Zeiger S. L. Musiek E. S. Zanoni G. Vidari G. Morrow J. D. Milne G. J. (2009). Neurotoxic lipid peroxidation species formed by ischemic stroke increase injury. *Free Radic. Biol. Med.* 47 1422–1431. 10.1016/j.freeradbiomed.2009.08.011 19699297 PMC2767385

[B105] Zhao C. Bateman A. (2015). Progranulin protects against the tissue damage of acute ischaemic stroke. *Brain* 138 1770–1773. 10.1093/brain/awv123 26106095

[B106] Zima T. Štípek S. Crkovská J. Němeček K. Pláteník J. Bártová V. (1996). Antioxidant enzymes-superoxide dismutase and glutathione peroxidase-in haemodialyzed patients. *Blood Purif.* 14 257–261. 10.1159/000170269 8738540

