# Supplementary figures and images for: Fourier-Transform Infrared Imaging Spectroscopy and Laser Ablation -ICPMS New Vistas for Biochemical Analyses of Ischemic Stroke in Rat Brain
Source: Front Neurosci. 2018 Sep 19;12:647. doi: 10.3389/fnins.2018.00647 (PMC6157330; doi:10.3389/fnins.2018.00647)

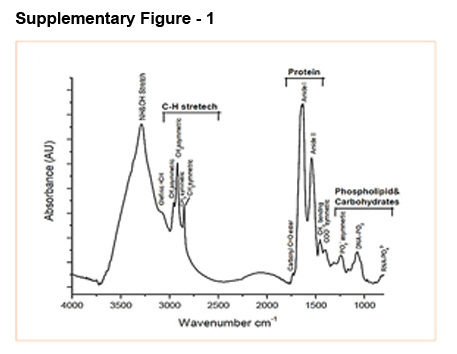

Supplement: FIGURE S1 — The representative FTIR spectrum of white matter (WM) in a native control rat brain in the spectral range of 4000–700 cm-1 and mechanical properties of the native and lesioned brain sections. Representative FTIR spectra of WM in the native control rat brain from region 4000–700 cm-1. [file Image_1.jpg]
