# Supplementary material for: Fourier-Transform Infrared Imaging Spectroscopy and Laser Ablation -ICPMS New Vistas for Biochemical Analyses of Ischemic Stroke in Rat Brain
Source: Front Neurosci. 2018 Sep 19;12:647. doi: 10.3389/fnins.2018.00647 (PMC6157330; doi:10.3389/fnins.2018.00647)
Supplement: TABLE S1 — Fourier transform Infrared (FTIR) spectral bands and their corresponding bio-chemical assignment. [file Table_1.pdf]

Supplementary Table - 1

Wavenumber (cm−1)	Assignment
3328	Amide A: N–H and O–H stretching vibrations of polysaccharides, proteins
3015	Olefin =CH stretching: unsaturated lipids, cholesterol esters
2960	CH_3 asymmetric stretching: mainly lipids with the little contribution from carbohydrates, proteins, nucleic acids.
2920	CH_2 asymmetric stretching: mainly lipids
2875	CH_3 symmetric stretching: protein side chains, lipids
2850	CH_2 symmetric stretching: mainly lipids
1720–1745	C=O stretching vibrations of lipids (triglycerides and cholesterol esters)
1710–1690	C=O asymmetric stretching: RNA and purine base
1705–1690	C=O asymmetric stretching vibrations: RNA, DNA
1654	Amide I: C=O (80%) and C–N (10%) stretching, N–H (10%) bending vibrations: proteins a-helix
1630–1640	Amide I: (80%) and C–N (10%) stretching, N–H (10%) bending vibrations: proteins β-structure
1610, 1578	C4-C5 and C=N stretching in imidazole ring of DNA, RNA 1515 Aromatic tyrosine ring
1540–1550	Amide II: N–H (60%) bending and C–N (40%) stretching vibrations:<br>proteins α-helix
1530	Amide II: N–H (60%) bending and C–N (40%) stretching vibrations:<br>proteins β-structure
1467	CH_2 bending vibrations: lipids and proteins
1455	CH_3 bending and CH_2 scissoring vibrations: lipids and proteins
1370–1400	COO- symmetric stretching and CH_3 bending vibrations: lipids,<br>Proteins
1330–1200	Amide III: proteins
1230–1244	PO^{2-} antisymmetric stretching vibrations: RNA, DNA and phospholipids
1090-1080	PO^{2-} symmetric stretching vibrations: RNA, DNA
1060, 1050	C–O stretching vibrations: deoxyribose/ribose DNA, RNA
996	RNA stretch and bend ring of uracil
965	PO_4^{3-} symmetric stretch (DNA) and deoxyribose-phosphate skeletal motions
